# Nosocomial Infections and Epidemiology of Antibiotic Resistance in Teaching Hospitals in South East of Iran

**DOI:** 10.5539/gjhs.v8n2p190

**Published:** 2015-06-25

**Authors:** Mahboobeh Rajabi, Mohammad Esmaeili Abdar, Hossein Rafiei, Mohammad Reza Aflatoonia, Zahra Esmaeili Abdar

**Affiliations:** 1 Health Services Management Research Center, Institute for Futures Studies in Health, Kerman University of Medical Sciences, Kerman, Iran; 2 Alborz University of Medical Sciences, Karaj, Iran; 3 Medical Surgical Department, Nursing and Midwifery School, Qazvin University of Medical Science, Qazvin, Iran; 4 Research Center for Tropical and Infectious Diseases, Kerman University of Medical Sciences, Kerman, Iran

**Keywords:** epidemiology, hospital infections, antibiotic resistance, developing country

## Abstract

**Aim:**

Antibiotic resistance as one of the most serious health threats worldwide leading to a high rate of morbidity and mortality. The aim of present study was to examine the prevalence of nosocomial infections (NIs) and pattern of antibiotic resistance in teaching hospitals in Iran

**Methods:**

This cross-sectional descriptive study was conducted in a period of one year in three teaching hospitals and all patients with suspected NIs symptoms were chooses. Among these patients who showed antibiotic resistance were included in the study. The samples for clinical test in laboratory were obtained with using standard methods and aseptic technique by trained personnel. Antibiotic susceptibility testing was performed by Kirby-Bauer’s disk diffusion method on Muller-Hinton agar (Hi Media, Mumbai, India) in accordance with the standards of the Clinical Laboratory Standards Institute.

**Results:**

During one year study, 561 patients with nosocomial infections were recognized and among them 340 patients (60.6%) showed some level of antibiotic resistance. The most common cause of NIs in present study was Acinetobacter and the most type of infection was respiratory system infections (52.7%). The highest resistance rate was against Ciprofloxacin (61.8%) followed by Imipenem (50.3%).

**Conclusion:**

Rate of NIs and antibiotics resistance is high in Iranian hospital. So Iranian health ministry should provide guideline and suitable programs for prevention of NIs and antibiotic therapy in hospitals.

## 1. Introduction

Nosocomial infections (NI), also known as hospital acquired infections, are global health problem which affect both developed and developing countries. Nosocomial infections are defined as infections occur within 48 hours after hospital admission, 3 days after discharge or 30 days after an operation (WHO, 2002). Prevalence of NIs are varies between 1.5 to 26.1 percent among different countries (Hashemi et al., 2010; Leaper et al., 2004; Kim et al., 2000; Gastmeier et al., 2001; Haley et al., 1985; Behnke et al., 2013; Petersen et al., 2010; Hughes et al., 2005; Nguyen et al., 2001; Balkhy et al., 2006; Saleem et al., 2013). Prevalence of NIs in Iranian hospitals reported between %1.3 to %10 (Hashemi et al., 2010; Askarian et al., 2012; Hajibagheri et al., 2006; Qorbanalizadehgan et al., 2008; Barak et al., 2012; Hojat et al., 2012; Hosseinrezaei et al., 2012). The increasing rate of NIs, causes more antibiotics usage which leads to economic burden, and as a final result, the increases rates of morbidity and mortality (Inweregbu et al., 2005; WHO, 2002; Hashemi et al., 2010). Despite international efforts to control NIs during past decades, NIs still remains a prevalent problem and one of patients antibiotic resistance causes in hospitals (Farr et al., 2001).

As the result of broad uses of antimicrobial agents, the infections pathogens have shifted to more resistant bacteria and this causes great problems for nosocomial infections control, prevention and treatment especially for hospital infections (Cars et al., 2005). Antibiotic resistance is a natural biological outcome of excess antibiotic uses which usually occurs when a microbe acquires a gene, which allows the microbe to inactivate the antibiotic activity or nullify its antimicrobial activity (Cars et al., 2005; Muto et al., 2003). The rapid development of antimicrobial resistance and its dissemination and burden become a serious public health worldwide, as WHO emphasized its importance on World Health Day 2011 (WHO., 2012). According to the CDC report, more than 70% of the bacteria causing NIs are resistant to at least one of the medications used to treat them (CDC, 2001). Also these infections have been more costly and more deadly than those are antibiotic susceptible strains of the same species (Muto, 2005).

There are many important affecting factors which may increase the risk of antibiotic resistance such as poor utilization of antimicrobial agents, transmission of resistant bacteria from patient to patient and from health care team members to patients, lack of guidelines for appropriate and judicious use of antimicrobial agents and lack of easy-to-use auditing tools for restriction and misuse of antimicrobial agents in the animal industry (Aly et al., 2012).

Although the resistance against antibiotics is high in Iranian hospitals, the pattern of this antimicrobial resistance has not been received enough attention. In a study performed in 2010, Ghadiri and colleagues reported that the most prevalent blood stream and urinary tract infections pathogens were Coagulase-negative staphylococci and Escherichia coli, respectively. The highest resistance rate of Coagulase-negative staphylococci and Escherichia coli was against Penicillin (91.1%) and Nalidixic acid (57.7%) (Ghadiri et al., 2012). In another study, Vahdani and colleagues investigated the prevalence of antibiotic resistance to Acinetobacter baumannii in a hospital in Tehran, Iran. They showed Acinetobacter baumannii resistance to Ceftazidime (96%), Ceftizoxime (95%), Ceftriaxon (93%), Amikacin (58%), Gentamicin (68%), Co-terimoxazole (85%), and Ciprofloxacin (85%) (Vahdani et al., 2011).

It is obvious that knowing about NI rate, the prevalent pathogens, and their antibiotic resistant pathogens is essential for effective decision-making on infection control policies, the rational formulation of public health care policies, the effective antibiotics therapy, the appropriate antibiotics usage guidelines and the national and international research agendas in this area, especially in countries with higher antibiotic use such as Iran. Although, broad-spectrum antibiotics are used for infection therapy especially for NIs in Iran, there is limited data on antibiotic resistance. So in the present study we examine the prevalence of NIs and pattern of antibiotic resistance in three teaching hospitals in southeast of Iran.

## 2. Materials and Methods

This cross-sectional descriptive study performed between January 2012 and January 2013 in three teaching hospitals (Afzalipoor, Shahid Bahonar and Shafa) in Kerman, a province in south east of Iran. These three hospitals provide the most important medical services of all parts of Kerman province. This project approval was obtained from both research center for Tropical and Infectious Diseases of Kerman University of Medical Sciences and the heads of the three mentioned hospitals prior to the study.

Inclusion criteria: All patients with nosocomial infections symptoms, who stayed more than 48 hour in hospitals and with no sign of bacterial colonization (like fever) at the time of admission, were chosen. Among these patients, the antibiotic susceptibility tests were done for those showed the symptoms of resistance to the prescribed antibiotic and patients with positive antibiotic resistance test were included in the study. Samples of blood, urine, sputum and wound secretion were obtained using standard methods and aseptic technique by trained personnel (physician and nurse). Antibiotic susceptibility testing also was performed by Kirby-Bauer’s disk diffusion method on Muller-Hinton agar (Hi Media, Mumbai, India) in accordance with the standards of the Clinical Laboratory Standards Institute (CLSI, formerly National Committee for Clinical Laboratory Standards [NCCLS]) guidelines (CLSI., 2008). The antibiotic concentration per disk was as follows: Amikacin, Amoxicillin, Ampicillin, Carbenicillin, cephalexin, Cefazolin, Cefepime, Cefixime, Cefuroxime, Cefotaxime, Ceftazidime, Ceftizoxime, Ceftriaxon, Cefuroxime, Cephalothin, Chloramphenicol, Ciprofloxacin, Clindamycin, Cloxacillin, Co-trimoxazole, Doxycycline, Erythromycin, Gentamicin, Imipenem, Kanamycin, Meropenem, Nalidixic acid, Nitrofurantoin, Oxacillin, Penicillin, Rancomycine, Tetracycline, Tobramycin and Vancomycin. All statistical analyses were performed using SPSS software (version 18.0; PASW Statistics). A variable was considered to be statistically significant if p < 0.05.

## 3. Results

During one year study, 561 patients with nosocomial infections were recognized. Of these 320 (57%) were male and 241 (43%) were female. The most of patients were in 21-30 years old, in Intensive Care Units and have respiratory system infections. Among 561 patients, 340 patients (60.6%) showed some level of antibiotic resistance. Of these 212 (62.4%) were male and 128 (37.6%) were female and most of them (22.1%) were between 21-30 years old. Patients who admitted to Intensive Care Units (ICU, CCU, NICU), both adults and neonatal, showed more antibiotics resistance compared to patients admitted to other wards. In terms of infection site, most infections were related to respiratory system infections (52.7%) followed by surgical site infection (31.5%), urinary tract infection (8.5%) and bloodstream infection (2.9%). About length of hospital stay, most of patients (51.2%) stayed between 5 and 30 days (Table 1). Among patients with antibiotic resistance, 84.4%, have had intravenous canola, 81.7%, urinary indwelling catheter, 59%, endotheracheal tube, 57.5%, mechanical ventilation, 24.8%, tracheostomy tube, 9.1%, arterial canola and 2.7% cerebral shunt. In addition, 65.5% of patients had at least one surgery. The most common cause of NIs in present study was Acinetobacter (%31.5), Klebsiella (%25.3), Pseudomonas (%13.2), Staphylococcus aureus (10%), Escherichia (7.4%) and Citrobacter (5%).

**Table 1:** Frequency of age, gender, length of stay in hospital, site of infection and admitted ward

Variables	Frequency	

	N	%
**Sex**		
Men	212	62.4
Women	128	37.6
**Age**		
1-10	15	4.4
11-20	48	14.1
21-30	75	22.1
31-40	41	12.0
41-50	31	9.1
51-60	44	12.9
61-70	37	10.9
71-80	24	7.1
81-95	25	7.4
**Length of stay in hospital**		
<5 days	26	7.6
5-30 days	140	51.2
>30 days	174	41.2
**Ward**		
ICU, CCU, NISU	217	63.8
Burn	56	16.5
Orthopedic	30	8.8
Neurology	19	5.6
Other Wards	18	5.3
**Site of infection**		
Respiratory system	179	52.7
Surgical site	107	31.5
Urinary tract	29	8.5
Bloodstream	10	2.9
Other	15	4.4

The highest resistance rate was against Ciprofloxacin (61.8%) followed by Imipenem (50.3%), and the lowest rate was against Penicillin, Amoxicillin, Rifampine and Ticarcillin (0.3%) (Table 2). Acinetobacter and Escherichia showed the most resistance to Ciprofloxacin and Klebsiella to Imipenem. Staphylococcus aureus showed the most resistance to Ceftriaxone, Pseudomonas to Cefazolin and Citrobacter to Gentamicin (Table 3).

**Table 2:** Frequency of resistance to different antibiotics

Antibiotics	Resistance %
Ciprofloxacin	61.8
Imipenem	50.3
Gentamicin	44.1
Amikacine	43.2
Ceftriaxon	39.7
Penicillin	0.3
Amoxicillin	0.3
Rifampin	0.3
Ticarcillin	0.3

**Table 3:** Prevalence of antibiotic resistance by bacteria

Antibiotics	Acinetobacter	Klebsiella	Staphylococcus aureus	Pseudomonas	Escherichia	Citrobacter
Amikacine	51.2	39.3	52.6	78.2	21.7	37.5
Cefazolin	30.5	40.9	15.4	85	41.7	37.5
Cefepime	36.2	21.3	12.8	15.6	4.5	6.3
Cefixime	6.3	11.6	63.1	25.1	34.8	18.8
Ceftazidime	25.8	17.2	5.1	37.6	4.5	12.5
Ceftriaxone	22.5	21.3	70.5	62.6	74.8	37.5
Cefuroxime	27.9	25.8	25.3	71.9	20	6.3
Ciprofloxacin	62	48.3	38.1	71.9	91	25
Gentamicin	29.5	39.3	48.4	46.9	90	43.8
Imipenem	51.9	49.4	25.6	78.2	46.1	31.3
Nalidixic acid	4.7	8	15.4	32.1	61.7	12.5
Co-trimoxazole	14.8	21.3	7.7	28.1	73.6	6.3
Cephalexin	3.1	3.4	17.9	0	13.6	6.3
Kanamycin	9.3	1.1	0	15.7	4.5	12.5

The results showed that the antibiotic resistance was more prevalent among men than women (p<0.05). The results also showed that resistance to some antibiotics such as Meropenem, Imipenem, Clindamycin, Ceftazidime, Cefepime and Amikacine was more prevalent in men and resistance to some other antibiotics such as Tobramycin, Chloramphenicol, Nalidocic acid and Methicilin was more prevalent among women. The findings also revealed that by increasing the length of hospital stay antibiotics resistance also increased (P<0.05). Patients in CCU, ICU, NICU and burning ward showed higher rates of antibiotics resistance in comparison with the patients of other wards. This difference was also statistically significant (P<0.05). The patients who had surgery, mechanical ventilation, cerebral shunt and Total Parenteral Nutrition (TPN) showed higher rates of antibiotic resistance, especially to Cefazolin, Cefteriaxone, Ceftizoxime and Cefotaxime (P<0.05).

## 4. Discussion

Since knowing the antibiotic resistance pattern is essential for appropriate therapy, we examined the distribution of antibiotic resistance in Kerman, Iran. During one year study, 561 patients with nosocomial infections were recognized. About 60.6% of patients with NIs showed some level of antibiotic resistance. The results showed that the antibiotic resistance was more prevalent among men than women (p<0.05) but in Ghadiri and colleagues study the antibiotic resistance was more prevalent among women. Most of patients were between 21-30 years old which could be due to preponderance of more NIs patients in this group. However, the young people in working age were more vulnerable to antibiotic resistance. Patients who admitted to Intensive Care Units (ICU, CCU, NICU) and burn ward showed more antibiotics resistance compared to patients admitted to other wards.

Factors like host, organism, and treatment may affect length of hospitalization with resistant organisms. In this study 51.2% of patients stayed between 5- 30 days at hospital. The findings consistent with some other studies showed antibiotics resistance had significant association with the length of hospital stay (Cosgrove, 2006; de Kraker et al., 2011).

The most used invasive devices were intravenous canola (84.4%) and urinary indwelling catheter (81.7%), and patients which had invasive devices treatments showed higher rates of antibiotic resistance. In Erbay and colleagues study in Turkey mechanical ventilation, nasogastric tube, tracheotomy and orogastric tube were found to be significant risk factors for nosocomial infection (Hakan Erbay et al., 2003). So to reduce nosocomial infections, minimizing the use of invasive devices and specific disinfection precautions during the application of invasive devices could be effective.

Most infections site was related to respiratory system (52.7%), surgical site (31.5%), urinary tract (8.5%) and bloodstream (2.9%). In Burns and colleagues study the most common infection sites were surgical site, pneumonia, urinary tract and bloodstream (Burns et al., 2012).

Results also showed that Acinetobacter and Klebsiella were the most common cause of NIs. This result is consistent with other studies in some developing countries in Latin America and South Africa (Sosa et al., 2010). Nevertheless in Aly and Balkhy study in six Persian Gulf Arab countries including Saudi Arabia, Qatar, Bahrain, Kuwait, Oman, and United Arab Emirates, the most prevalent determined microorganisms were Escherichia coli, Klebsiella, Pneumoniae, Pseudomonas aeruginosa, MRSA and Acinetobacter (Aly et al., 2012). In Erbay and colleagues study Pseudomonas aeruginosa, Staphylococcus aureus and Acinetobacter were the frequent microorganisms (Hakan et al., 2003).

Highest resistance rate was against Ciprofloxacin (61.8%) followed by Imipenem (50.3%), and the lowest rate was against Penicillin, Amoxicillin, Rifampin and Ticarcillin (0.3%). Acinetobacter and Escherichia showed the most resistance to Ciprofloxacin, Klebsiella to imipenem, staphylococcus aureus to Ceftriaxon, Pseudomonas to Cefazolin and Citrobacter to Gentimicin. Zamini and colleagues study showed that Acinetobacter and Escherichia had the most resistance to Ciprofloxacin and Ceftriaxone (Zamani et al., 2014). In Aly and Balkhy investigation the Enterobacteriaceae and Staphylococcus aureus had resistance to broad spectrum of third generations of cephalosporins and flucloxacillin (i.e., methicillin-resistant Staphylococcus aureus or MRSA) (Aly et al., 2012). Hashemi and colleagues identified the prevalence of antibiotic resistance of Entrobacteriaceae strains isolated from hospital-acquired and community-acquired infections in west of Iran. Highest and lowest rate of resistance were related to Ampicillin (98.8%) and Piperacillin (3.7%). They also showed that most isolates have resistant to Cefazolin, Cefixime, and Co-trimoxazole (Hashemi et al., 2013).

In some other studies several reasons have demonstrated for nosocomial infections and antimicrobial resistance and many of those reasons exist in Iranian hospitals. These reasons including readily available broad spectrum antibiotics (such as 3^rd^ and 4^th^ generations of Cephalosporins, Quinolones and Carbapenems), lack of antimicrobial usage guidelines and programs (especially in the hospitals where broad spectrum of antimicrobial agents are using), lack of new and modern hospitals with suitable architectural designs and enough space for proper isolation of infected and colonized patients with multi-drug resistant organisms, lack of intensive system and trained and educated personnel for infections control in hospitals and lack of clinical pharmacologist in the field of infectious diseases (Goldman and Huskins., 1997, Aly et al., 2012). Furthermore, lack of staff to patients ratio (specially nurses to patients ratio in Iran), lack of information about prevalence and incidence of antibiotic resistance, use of antibiotics by Iranian people without prescription of physician, availability of most of antibiotics in drugstores for people without needs to physicians order (over-the-counter) in Iran, are very important and influential.

## 5. Conclusion

Similar to other developing countries, NIS and antimicrobial resistance has not received enough attention in Iran. Results of present study like some other studies showed that the rate of NIs and antibiotics resistance is high in Iranian hospitals. Therefore Iranian health ministry should provide guideline and suitable programs for prevention of NIs and antibiotic therapy in hospitals especially using infection specialist beside the other physicians in hospitals for prescribing appropriate antibiotics, which can prevent the antibiotic resistance to some extent and leads to better treatment and sooner discharge of the patients. Moreover, educational and medical systems in Iran need to consider training of some well-educated personnel on the prevention and management of NIs and antibiotic resistance. Further research needed in this regards in Iran.
